# Clinical Evolution of Tardive Cervical Dystonia from Antecollis to Retrocollis

**DOI:** 10.7759/cureus.38565

**Published:** 2023-05-05

**Authors:** Octávia Costa, Sara Varanda, Gisela Carneiro, Ana Margarida Rodrigues

**Affiliations:** 1 Neurology Department, Hospital de Braga, Braga, PRT

**Keywords:** antipsychotic medication, alzheimer’s dementia, cervical dystonia, olanzapine side effects, tardive dystonia

## Abstract

Tardive dystonia occurs after exposure, over months to years, to antipsychotics and other drugs that block dopaminergic receptors. Anterocollis is a rare form of cervical dystonia which is usually disabling for the patient.

Here, we present the case of a 61-year-old woman with Alzheimer’s dementia diagnosed eight years ago who was previously medicated with antipsychotics. Two years before admission, she was medicated with olanzapine. She presented to the emergency room with a sustained flexion posture of the neck that was difficult to feed. She had a marked and fixed anterocollis and severe akathisia. After the administration of propofol to perform computerized tomography, the abnormal posture disappeared. Subsequently, she was started on biperiden without improvement. One week later, olanzapine was suspended, and she was progressively started on propranolol, trihexyphenidyl, and tetrabenazine. Cervical posture improved, but two weeks later, she presented with a left laterocollis, which allowed feeding, and improvement of akathisia.

We present a case of tardive dystonia supported by the beginning of dystonia five months after olanzapine administration and improvement after its suspension. The coexistence of degenerative pathology is a risk factor for dystonia, which often persists despite the suspension of the causative agent. Therefore, non-pharmacological treatment and approach with antipsychotics with a better profile of extrapyramidal effects should be preferred in patients with dementia.

## Introduction

The tardive syndrome consists of a group of late-onset and usually persistent involuntary movements caused by long-term dopamine receptor-blocking agents [1]. These extrapyramidal syndromes include different manifestations such as dyskinesia, stereotypies, dystonia, akathisia, myoclonus, tremor, tics, and pain.

The pathophysiology of tardive dystonia (TD) is proposed to result from chronic blockade of dopamine receptors (particularly D2 and possibly D3 receptors), which induces upregulation of D2 receptors and causes postsynaptic dopamine receptor hypersensitivity. D2 receptors are inhibitory receptors that inhibit the inhibitory indirect pathway, hence, their hypersensitivity produces hyperkinetic movements. Serotonin receptors (5-HT2 receptors, in particular), which are widely distributed in the striatum, interact with dopaminergic neurotransmission, and their blockade reduces D2 receptor upregulation. This mechanism explains why second-generation antipsychotics, which present a lower D2 receptor affinity but higher 5-HT2 receptor affinity, have a lower risk of inducing TD than first-generation antipsychotics [2,3].

Some well-established risk factors for TD include older age, female sex, white or African descent, genetic variants involving antipsychotic metabolism and dopamine function, longer disease duration, and preexisting mood disorders [2]. TD prevalence is variable, ranging between 0.4 and 5%, and is associated with younger age and slight male predominance when compared with the other extrapyramidal syndromes [2,4].

TD occurs after months to years of exposure to antipsychotics and other therapeutics with the ability to block dopaminergic receptors [4,5]. The most frequently involved muscles are cervical and cranial, provoking dysphagia and impairing communication. The initial presentation as anterocollis is a rare form of cervical dystonia that can change over time [4,6].

Here, we report an uncommon case of TD after olanzapine exposure, an atypical antipsychotic, with a severe commitment to feeding and changing presentation throughout evolution.

## Case presentation

A 61-year-old woman presented with Alzheimer’s disease eight years ago. She had no other relevant diseases in the past and no known prior medication. Moreover, there was no history of neurological or psychiatric diseases in her family.

She was submitted to an experimental deep brain stimulation surgery five years ago, but the electrodes were turned off after one year due to the absence of any benefit. No side effects, including movement disorders, were noticeable. During the follow-up, she was medicated with rivastigmine 9.5 mg TD. As she displayed a behavior change, predominantly characterized by agitation, which predominated in the evening, she was successively medicated with clozapine 25 mg twice a day, followed by mirtazapine 15 mg, and levomepromazine 25 mg. These interventions were not successful in behavior control.

She again presented to the emergency department with psychomotor agitation. She had a right palmomental reflex, and her speech was not fluent, with paraphasias, neologisms, and akathisia. At this point, the above-mentioned antipsychotics were stopped and olanzapine 10 mg at night was initiated.

**Figure 1:**
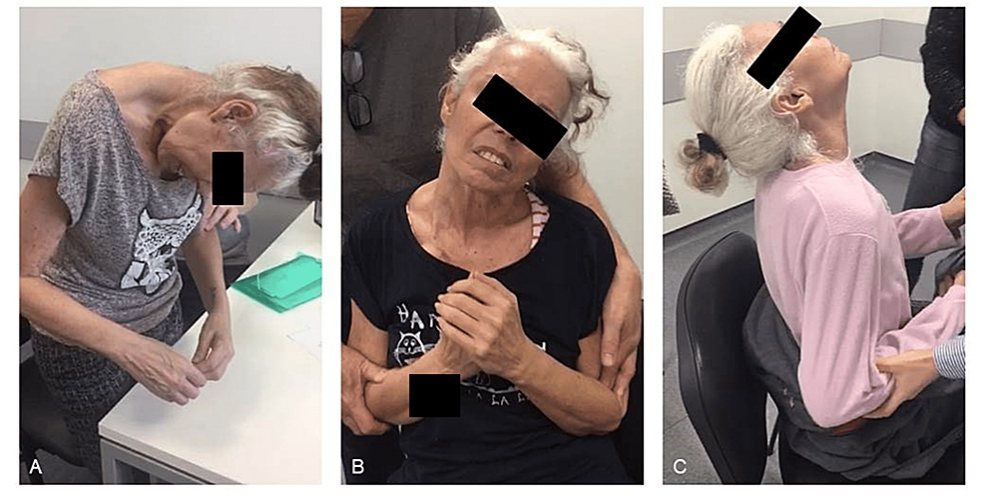
The patient showing marked antecollis six months after olanzapine prescription (A). Three weeks after the suspension of olanzapine, the pattern of dystonia was modified, presenting as left laterocollis (B). The pattern of dystonia modified to retrocollis (C).

**Figure 2:**
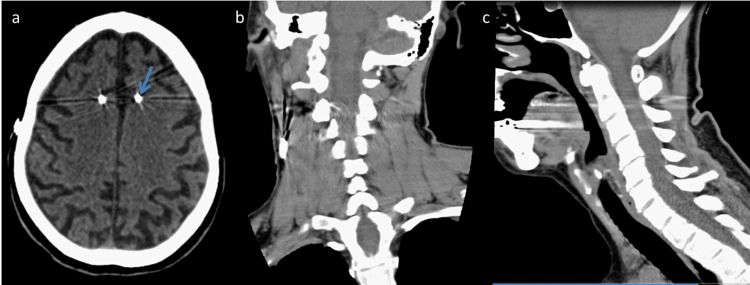
Computerized tomography (CT) of the brain showing deep brain stimulation electrodes (blue arrow) and generalized cortical atrophy (a). Cervical CT showing no structural causes of abnormal posture (b, c).

Six months later, she returned to the hospital with a painful involuntary anterior neck flexion which started three weeks before. This posture made non-verbal communication and feeding challenging. On examination, she presented marked anterocollis and pain during manipulation attempts (Figure 1, Panel A). The analytical study was normal. Cerebral and cervical computerized tomography (CT) showed generalized cortical atrophy (Figure 2). There was posture normalization during propofol administration needed to perform the examination, which made a structural cause unlikely.

Given that the antipsychotic drug was modified a few months earlier, our main hypothesis was TD secondary to olanzapine. The drug was gradually reduced until its suspension, and lorazepam and thiocolchicoside were prescribed.

One week later, she maintained akathisia and anterocollis, following which propranolol and trihexyphenidyl 2mg three times a day were initiated.

Two weeks later, the patient showed improvement in akathisia, but maintained psychomotor agitation and modification of dystonia pattern, presenting as left laterocollis. At this time, she was prescribed quetiapine 50 mg and tetrabenazine 25 mg (Figure 1, Panel B).

Four months later, the pattern changed to retrocollis (Figure 1, Panel C). By this time, it was decided to apply botulinum toxin A on the right and left splenium muscles (80+80 U) with a slight improvement in posture.

## Discussion

We present a case of TD after olanzapine exposure in a patient suffering from Alzheimer’s disease. TD is a rare and usually late complication of the use of dopamine receptor-blocking drugs, including first-generation antipsychotics (such as chlorpromazine, haloperidol, fluphenazine, thioridazine, and pimozide) and prokinetic drugs such as metoclopramide [1]. This entity is less common in individuals taking second-generation antipsychotics. Trugman et al. proposed that TD mechanism consists of repetitive stimulation of the D1 receptor by endogenous dopamine, resulting in sensitization of the D1-mediated striatal output in the presence of D2 receptor blockade [7].

Burke et al. defined TD as including the following criteria: (1) presence of chronic dystonia, (2) history of antipsychotic drug treatment, (3) exclusion of known causes of secondary dystonia, and (4) negative family history for dystonia [1].

Remarkably, our case is an example of the change in the pattern of dystonia over the course of the disease, as previously reported by Kiriakakis et al. [4]. We intend to highlight the change in the dystonia pattern with the adoption of a very exuberant retrocollis posture that greatly limited the patient’s quality of life, even after the cessation of the antipsychotic.

Although in most cases the typical presentation of cervical TD is retrocollis, in this case, we show an uncommon initial presentation in the form of a marked antecollis limiting the patient’s daily routine [4,6].

Notably, in this case, TD appeared after the use of olanzapine, although few cases were reported using this drug. The pathological and physiological mechanism of olanzapine-induced TD is not clear. According to Trugman et al., olanzapine may cause TD by persistent inhibition of dopamine neurotransmitters leading to overly sensitive postsynaptic dopamine receptors [7]. In comparison with typical antipsychotics, olanzapine saturates serotonin type 2 receptors and demonstrates a higher serotonin type 2 than D2 receptor occupancy at all doses [3]. Moreover, olanzapine is thought to act more at mesolimbic than at the nigrostriatal pathway [5].

The pathogenesis of TD may be multifactorial and probably includes individual susceptibility [1,4]. The pathophysiology of TD is proposed to result from chronic blockade of dopamine receptors, predominantly D2 receptors, which induces upregulation of D2 receptors and causes postsynaptic dopamine receptor hypersensitivity. D2 receptors are inhibitory receptors expressed on striatal medium spiny neurons that inhibit the inhibitory indirect pathway, hence, their hypersensitivity produces hyperkinetic movements [2].

Our patient was at an increased risk to suffer an extrapyramidal syndrome such as TD, hence, the previous diagnosis of neurodegenerative disease, such as Alzheimer’s disease [4].

The patient also presented tardive akathisia, which is a neurodegenerative pathology already described as a risk factor associated with antipsychotic intake [5]. Our patient represents an uncommon association between two different extrapyramidal syndromes, akathisia and TD. A previous study showed that their combination has a prevalence of about 1% [8]. This study also demonstrated a relationship between the various extrapyramidal syndromes, particularly in psychiatric patients [8]. These data remind us that when an extrapyramidal syndrome is present, we must monitor for the presence of another one.

Considering that remission is rare even with causal agent suspension and that the best treatment is still debated, it is crucial to be careful in the moment of choosing an antipsychotic agent and trying to balance antipsychotic potency and extrapyramidal effects [1,4,6,9]. The best medical treatment of TD has been described as limited but the first step in treatment is discontinuation of antipsychotics [10]. If discontinuation is not possible, it is acceptable to change from first-generation antipsychotics to second-generation antipsychotics. Clozapine is the preferred agent to treat TD. Some studies have shown a significant benefit of using this drug [7,11-13]. The ability of clozapine to treat TD may be related to D1 receptor antagonism [12]. Clozapine has a higher affinity for D1 and a lower affinity for D2 dopamine receptors. Furthermore, some studies reported benefit with olanzapine [14], quetiapine [15], and aripiprazole [16].

The VMAT2 inhibitors (such as tetrabenazine) are dopamine-depleting medications that act by inhibiting the transport and sequestration of monoamines into presynaptic vesicles, therefore, promoting monoamine degradation in the cytosol and reducing dopaminergic transmission. Studies have previously demonstrated improvement in TD by using this drug with dosages of up to 250 mg [1,4]. One of the first descriptions showed improvement in 68% of patients treated [1]. Anticholinergics such as trihexyphenidyl with dosages between 6 and 36 mg achieved some benefit [1,4]. Furthermore, benzodiazepines such as clonazepam were previously reported to improve TD [17].

Botulinum toxin A [18] injected into the contorted muscles causes a permanent blockage of neurotransmission at the motor endplates by inhibiting acetylcholine release from nerve endings, causing a marked improvement that lasts only a few months. Kiriakakis et al. reported improvement in 83% of patients with tardive cervical dystonia [4].

Baclofen, provoking activation of GABAb receptor, was responsible for mild improvement in 56% of patients in retrospective studies. It can be administered intrathecally in severe cases [2].

In our case, we tried different drugs. Quetiapine was gradually introduced and used as an antipsychotic, permitting a sustained improvement of the patient’s dystonic symptoms, without loss of psychotic symptom control. Tetrabenazine was a useful drug for dystonia control, but the most favorable results were achieved by applying botulinum toxin A.

Deep brain stimulation of the globus pallidus pars interna is an established treatment for severe TD refractory to medical treatment [19]. In addition, deep brain stimulation of the subthalamic nucleus has been reported as beneficial in some patients [20].

## Conclusions

We present a case of TD supported by the beginning of dystonia five months after olanzapine administration and improvement after its suspension. TD is a rare and usually late complication of the use of dopamine receptor-blocking drugs.

The coexistence of degenerative pathology is a risk factor for dystonia, which often persists despite the suspension of the causative agent. Our case represents an impressive example of TD pattern modification throughout time and causing an important limitation on daily activities such as feeding.

Therapeutic management is usually difficult and requires neuroleptic discontinuation, if possible. This first step is not often feasible in psychiatric patients. In that case, the antipsychotic must be changed for a better drug, such as clozapine. If no improvement is seen, other drugs should be tried such as VMAT2-inhibitors, anticholinergic drugs, clonazepam, and baclofen. In focal dystonia, botulinum toxin treatment can be useful. Furthermore, deep brain stimulation has shown some improvement. We suggest that antipsychotics with a better profile of extrapyramidal effects such as clozapine should be preferred in patients with dementia.
